# Hydrogel Patterns in Microfluidic Devices by Do-It-Yourself UV-Photolithography Suitable for Very Large-Scale Integration

**DOI:** 10.3390/mi11050479

**Published:** 2020-05-02

**Authors:** Anthony Beck, Franziska Obst, Mathias Busek, Stefan Grünzner, Philipp J. Mehner, Georgi Paschew, Dietmar Appelhans, Brigitte Voit, Andreas Richter

**Affiliations:** 1 Institut für Halbleiter- und Mikrosystemtechnik, Technische Universität Dresden, 01187 Dresden, Germany; Anthony.Beck@tu-dresden.de (A.B.); mathias.busek@medisin.uio.no (M.B.); stefan.gruenzner1@tu-dresden.de (S.G.); philipp_jan.mehner@tu-dresden.de (P.J.M.); georgi.paschew@tu-dresden.de (G.P.); 2 Leibniz-Institut für Polymerforschung Dresden e.V., Hohe Straße 6, 01069 Dresden, Germany; obst@ipfdd.de (F.O.); applhans@ipfdd.de (D.A.); voit@ipfdd.de (B.V.); 3 Chair Organic Chemistry of Polymers, Technische Universität Dresden, 01062 Dresden, Germany

**Keywords:** very large-scale integrated (VLSI), miniaturization of hydrogel structures, poly(*N*-isopropylacrylamide) (PNIPAAm), poly((ethylene glycol) diacrylate) (PEGDA), polydimethylsiloxane (PDMS), hydrogel integration methods, in situ photopolymerization, photolithography, microfluidics, enzymatic microreactor, bioactive hydrogels

## Abstract

The interest in large-scale integrated (LSI) microfluidic systems that perform high-throughput biological and chemical laboratory investigations on a single chip is steadily growing. Such highly integrated Labs-on-a-Chip (LoC) provide fast analysis, high functionality, outstanding reproducibility at low cost per sample, and small demand of reagents. One LoC platform technology capable of LSI relies on specific intrinsically active polymers, the so-called stimuli-responsive hydrogels. Analogous to microelectronics, the active components of the chips can be realized by photolithographic micro-patterning of functional layers. The miniaturization potential and the integration degree of the microfluidic circuits depend on the capability of the photolithographic process to pattern hydrogel layers with high resolution, and they typically require expensive cleanroom equipment. Here, we propose, compare, and discuss a cost-efficient do-it-yourself (DIY) photolithographic set-up suitable to micro-pattern hydrogel-layers with a resolution as needed for very large-scale integrated (VLSI) microfluidics. The achievable structure dimensions are in the lower micrometer scale, down to a feature size of 20 µm with aspect ratios of 1:5 and maximum integration densities of 20,000 hydrogel patterns per cm². Furthermore, we demonstrate the effects of miniaturization on the efficiency of a hydrogel-based microreactor system by increasing the surface area to volume (SA:V) ratio of integrated bioactive hydrogels. We then determine and discuss a correlation between ultraviolet (UV) exposure time, cross-linking density of polymers, and the degree of immobilization of bioactive components.

## 1. Introduction

Analytical Labs-on-a-Chip (LoC) systems can be broadly divided into two directions of application. Point-of-care tests enable fast, cost-efficient analysis regardless of location and expensive lab equipment like in paper microfluidics. High-throughput LoCs are intended to reduce costs per sample, processing time, and the demand of samples and chemicals while increasing the output quality and reliability of analytical tests through system miniaturization, high integration of active components, and parallelization of specific reaction protocols [1].

The realization of highly integrated LoCs for high-throughput analyses is challenging. Firstly, there is a need for a powerful concept providing large address space to control a large number of microfluidic components individually. Secondly, fabrication technologies are required to realize a high density of channels and active components on chip substrates whose area is typically large compared to microelectronic integrated circuits. There are only three LoC platforms with demonstrated large-scale integration (LSI, hundreds to several thousand active components per chip) capabilities. The most important and the only commercially successful high-throughput LoC platform is pneumatic microfluidics. This concept is based on two inventions. The multi-layer soft lithography allows the realization of a large number of active basic components, which are membrane valves, on one substrate [2]. Pneumatic multiplexers significantly reduce the number of electro-pneumatic control channels making them suitable for LSI [3]. The fabrication technology already reached the level of very large-scale integration (more than 10,000 to 100,000 active components per chip) [4]. However, most LSI applications of pneumatic microfluidics use controllers with only two electro-pneumatic channels controlling a few, typically two, independent compartments, each consisting of thousands of valves for massive parallelization of analytical protocols [5]. The second platform is the electro-wetting on dielectric (EWOD) hybrid technology. EWOD is able to manipulate droplets on large and freely programmable electrode arrays fabricated with common thin-film processes of silicon-based microsystems technology, which are controlled by electronic drivers [6]. However, their application may be more directed to the advantages of a freely programmable microfluidic platform than to high-throughput analyses. The third approach is based on polymeric active material components able to drastically change their volume, stiffness, and consistence. Temperature-sensitive hydrogels with lower critical solution temperature characteristics are the active-material base of microelectromechanical system (MEMS)-type LoCs. Their swelling degree and, thus, their volume can be controlled via electro-thermal electrode interfaces. In LoC technology, this platform provides a versatile choice of active components including micro-valves [7], active sphincters [8], micro-pumps [9], micro-chemostats [10], storage [11], filters [12], and enzymatic reaction spaces [13,14]. The LSI control is realized using opto-electro-thermic controllers [15].

Polymeric active-material components are also the base for another basic concept of LoC technology called chemo-fluidics. Chemo-fluidic integrated circuits are self-controlling and self-powered LoC systems which significantly reduce the need for external equipment. Their active material components are completely controlled by the fluid’s chemical content, and they also take their fuel from the fluid inside the microfluidic channel. Basic components of chemo-fluidics are chemo-fluidic switches for disposable LoCs [16] and chemo-fluidic transistors [17] for reusable systems. Analytical protocols can be programmed via hardware instructions including logic gates, oscillators [18] and flipflops [19]. However, chemo-fluidics technologies are in their infancy, and several challenges have to be mastered. 

One crucial challenge for both hydrogel platforms, MEMS and chemo-fluidics, is to integrate a large number of hydrogel components with high integration densities on the channel-containing substrates that have typically large areas. From a technical point of view, hydrogels are very unusual and difficult materials. They are the softest technical solid-state materials; they are only slightly cross-linked, porous polymers leading to issues with film homogeneity, which drastically changes their properties with complex thermodynamics and swelling kinetics [20,21]. Standard manufacturing methods typically result in inadequate reproducibility and homogeneity of hydrogel component properties over the substrate area not matching the requirements of quantitative analysis, especially after miniaturization [22]. 

Nevertheless, various methods were developed to fabricate hydrogel patterns on substrates with feature sizes down to the lower micrometer range for high integration densities, high aspect ratios, and various substrate materials [23,24,25]. While inkjet three-dimensional (3D) printing is suited for the millimeter range [26], smaller hydrogel structures can be realized by various photolithography techniques based on photomasks or direct writing processes. Mask-based photo-cross-linking of dried polymer films allows structure resolution down to one micron, whereas the free-radical ultraviolet (UV) polymerization from a monomer solution is well suited for patterns down to 20 µm [27,28,29]. Direct writing multiphoton lithography allows patterning in the lower micron range [30]. Very high integration densities (4.4 × 10^7^ per cm²) of hydrogel columns with an aspect ratio of 1:12 were achieved using a replica molding technique from partially polymerized precursor solution, which was completed by a photo-induced cross-linking process [23]. However, patterning of hydrogel layers with resolution smaller than 40 µm usually requires expensive industrial photolithographic equipment with an additional investment of at least 50,000 €.

In this work, we present a do-it-yourself (DIY) UV photolithographic station with overall material costs of 5000 € and similar photolithographic benchmarks in terms of illumination conformity, parallelization, and homogeneity of UV light intensity at a large circular illumination area of 90 mm diameter. Initially, we discuss its performance on the hydrogel poly(*N*-isopropylacrylamide) (PNIPAAm), which we use in different compositions as an actuating, chemo-fluidic, or sensor component in microfluidic systems [16,19,31]. PNIPAAm is a classic stimuli-responsive or smart hydrogel with volume phase transition behavior, which was continuously investigated since the 1980s. A second hydrogel type we investigate is poly((ethylene glycol) diacrylate) (PEGDA), which is well suited as a matrix for cell cultivation or to immobilize enzymes because of its biocompatibility and softness [13]. This work addresses the evaluation of suitable technologies for effective (fast, adaptable, and cost efficient) production of microfluidic devices with integrated photopolymerized hydrogels, while the focus is on the accurate and reproducible structure transfer during miniaturization. 

## 2. Materials and Methods 

### 2.1. Photolithographic Set-Up

**Figure 1:**
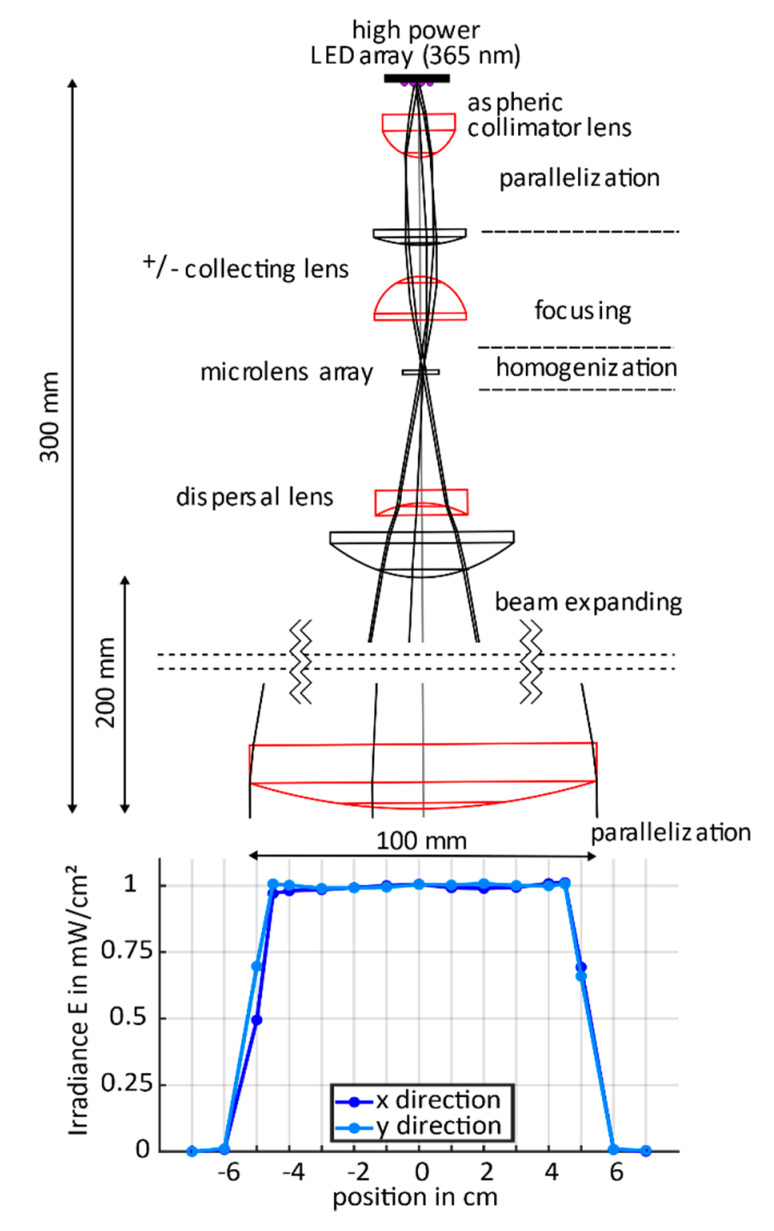
Top: Arrangement of the lenses in the lithography setup (true to scale). Bottom: Profile for the distribution of ultraviolet (UV) intensity at 365 nm wavelength measured over 16 cm in *x*- and *y*-direction across the light spot.

A 4 × 5 UV light-emitting diode (LED) array (CBM-120 Mosaic, Luminus Inc., Sunnyvale, CA, USA) was chosen as the light source, enabling high power density and uniform emission at a wavelength of 365 nm (i-line). For constant light source parameters, active air or water cooling of the LED-array backside is necessary. Figure 1 (top) shows the schematic of the beam path and the lens set-up, which is mounted on an adjustable rail system (Thorlabs Inc., New Jersey, USA) with total dimensions of 30 × 20 × 60 cm^3^ (length, width, height).

The light emitted by the LED array is firstly collected and then parallelized by an aspheric collimator. Subsequently, the light is focused by a collector lens on a microlens array (10 × 10 mm², 300 µm pitch, and 0.5° divergence angle, Edmund Optics^®^, Barrington, NJ, USA). Here, most of the homogenization of the incident beam is achieved by splitting it into an array of overlapping beams. The use of only one microlens array is relatively simple from a practical and user perspective. Higher homogenization can be achieved with an additional microlens array coupled in-line, but this also requires much more adjusting effort [32,33].

After homogenization, the beam is expanded by a combination of a dispersal and a plano-convex lens. Finally, the beam is parallelized again with a large field lens (100 mm diameter) to a visible spot size of 12 cm. The light intensity distribution (Figure 1 (bottom)) is measured with a UV radiometer calibrated at a wavelength of 365 nm (PLC UVA+ Sensor, Opsytec Dr. Groebel GmbH, Ettlingen, Germany) in the *x*- and *y*-direction of a total circular area of 16 cm diameter. With an intensity of 1 mW/cm², a flattop profile with a homogeneity of 98.98% over a circular area within 9 cm in diameter was achieved (empirical standard deviation of 1.02%), which is optimized for the three-inch or even four-inch wafer technology. The in-line photolithography set-up presented here, including the light source, various lenses, and opto-mechanics, ranks at an overall cost of 5000 €. Compared with commercial photolithographic mask aligners of at least 50,000 € providing similar benchmarks, our photolithographic system is very cost-efficient. Additional information on the software for beam path prediction for the development of the photolithography system and more details on the optic components are given in Appendix A.

### 2.2. Preparation of the Hydrogel Precursor

Before preparing the monomer solution for PNIPAAm gels, *N*-isopropylacrylamide (NIPAAm, Sigma-Aldrich, St. Louis, MO, USA) was recrystallized with n-hexane (VWR, International GmbH, Darmstadt, Germany) for purification purposes. For the monomer solution, NIPAAm (689.6 mg), the cross-linker *N,N*’-methylene-bis-acrylamide (24.1 mg BIS, Sigma-Aldrich, St. Louis, MO, USA) and the photoinitiator lithium phenyl-2,4,6-trimethyl-benzoylphosphinate (18.4 mg LAP, Bio-Techne GmbH, Wiesbaden, Germany) were mixed in deionized water (5 mL) under exclusion of light in a flask and stirred until all solids were dissolved. Then, the solution in the sealed flask was purged with argon to remove oxygen, thereby limiting its influence on the free radical polymerization later on. As previous investigations showed, the homogeneity of photostructured PNIPAAm gels increases upon handling the process under inert Ar atmosphere in a glove box [34]. 

### 2.3. Photopolymerization Process

**Figure 2:**
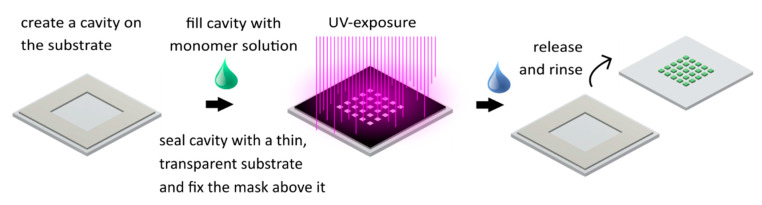
Standard photopolymerization procedure of hydrogel structures on various substrates.

In Figure 2, the photopolymerization process of a hydrogel array on a substrate is shown. The first step is to create a cavity on a clean substrate (glass or plastic), which will be the polymerization chamber, whereas the height of the walls defines the height of the hydrogel structures. Here, a spin-coated polydimethylsiloxane (PDMS) layer with the desired defined height was cut into the shape of a frame and used as spacer material. Subsequently, the polymerization chamber was filled with the prepared monomer solution. The cavity was sealed with a thin glass slide or UV transparent plastic foil and a photomask was adjusted above. Subsequently, the solution was exposed to UV light for 15 s with the presented recipe. After removing the mask and the thin substrate on top of the cavity, the remaining monomer solution was rinsed with isopropyl alcohol (IPA) and deionized water from the substrates. The adhesion of the gel is sufficient as long as the polymerization process takes place under exclusion of oxygen (e.g., substrates incubated in Ar)Ar-atmosphere for at least 24 h). Additional recommendations on hydrogel structuring process and materials can be found in Appendix B.

### 2.4. Manufacturing of the Microfluidic Systems and Hydrogel Integration

The microfluidic structures were produced by soft lithography [35]. PDMS was poured on a master, which was the negative of the microfluidic-channel network. The master was developed from a photolithographically exposed negative resist. Depending on the structure size, different resist systems can be used. The dry film resist (Elga Europe s.r.l, Milan, Italy) is suitable for structures down to the lower micrometer range. For sub-micrometer feature sizes, SU-8 (NanoTM MicroChem, Kayaku, Westborough, MA, USA) can be used. Next, PDMS was poured over the master and baked for 12 h at 40 °C (lower temperatures are preferred to reduce shrinking) [36]. Then, the PDMS-chip was removed from the master and plasma-bonded to a glass slide. For this, we used a low-pressure plasma cleaner (Zepto, Diener Electronic GmbH & Co. KG, Ebhausen, Germany). Oxygen plasma treatment was done for 45 s at medium vacuum (<102 Pa) with an oxygen influx of 80 l_n_/h and 15 W output power at 40 kHz frequency.

**Figure 3:**
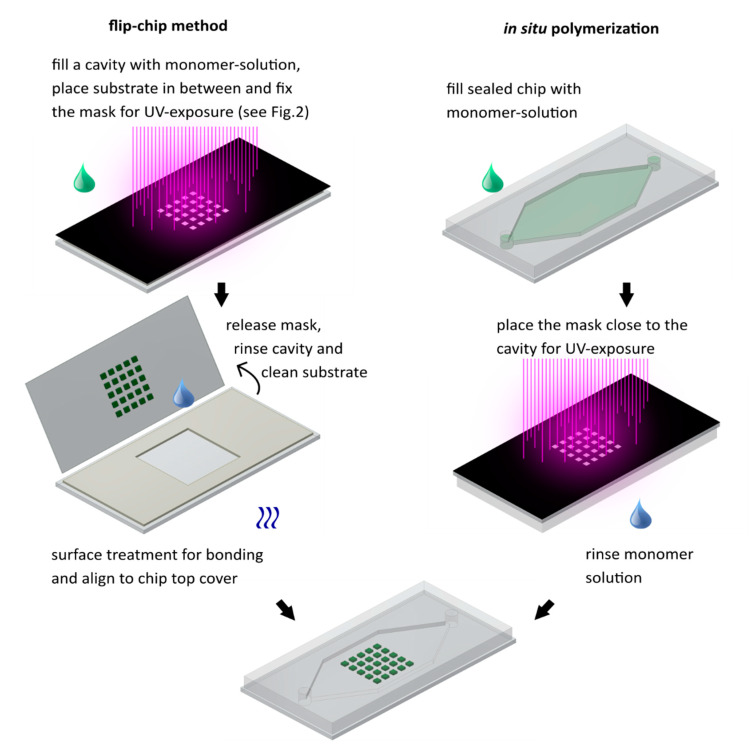
The flip-chip method (**left**) and in situ polymerization (**right**). Both methods result in a microfluidic chip with multiple integrated hydrogels.

For the fabrication of hydrogel-based microsystems, two large-scale integration concepts are established: the flip-chip technology and in situ polymerization [37,38]. An overview of the procedures is given in the illustrations of Figure 3. On the left, the process of the flip-chip method is illustrated, and, on the right-hand side, in situ polymerization is illustrated.

#### 2.4.1. Flip-Chip Method

Firstly, a cavity was created on a substrate corresponding to the size of the chip (comparable procedure to that shown in Figure 2). Again, the cavity defined the height of the hydrogels. The cavity was filled with the prepared monomer solution, sealed with a thin glass substrate, and exposed to UV light through a mask. After exposure, the mask and the thin substrate with the hydrogels were removed from the cavity, and the remaining monomer solution was rinsed with IPA and DI water. Thereafter, the substrate surface with the structured hydrogels and the matching microfluidic chip were activated by oxygen plasma treatment. By precisely aligning the channel structures with the corresponding hydrogel structures and bonding both substrates, a closed microfluidic chip with integrated hydrogels was fabricated.

#### 2.4.2. In Situ Polymerization

To integrate hydrogels by in situ polymerization, a plasma-bonded PDMS-on-glass microfluidic chip was prepared and filled with an Ar rinsed monomer solution. After aligning and fixing the mask to the bottom side of the microfluidic chip, the solution was exposed through the mask, and hydrogels were polymerized in place. Finally, the remaining monomer solution was completely flushed out of the system. In the case of PNIPAAm, the chip was released from the mask and placed in a thermal bath. As a result, the created hydrogels collapsed (shrank) and the residual monomer solution could be easily rinsed out through the chip inlets by hand with a syringe or with a connected pump. When polymerizing non-temperature-sensitive hydrogels like PEGDA, there has to be enough space in the channels for the monomer solution to get flushed out.

### 2.5. Preperation of Hydrogel-Based Enzymatic Microreactors

Catalytic activities of non-immobilized enzymes, glucose oxidase from *Aspergillus niger* (GOx) and horseradish peroxidase (HRP), were determined in a 2,2′-azino-bis(3-ethylbenzothiazoline-6-sulfonic acid) diammonium salt (ABTS) assay as previously reported [13,14]. For the production of microfluidic reactors, we used the in situ polymerization method instead of the flip-chip method to avoid impacts of plasma activation and vacuum treatment on the enzyme activity. The microfluidic chip was filled with a precursor solution consisting of PEGDA (1000 mg, 57 mol.%), 2-(dimethylamino)ethyl methacrylate (DMAEMA, 101.4 µL, 27 mol.%), 2-hydroxyethyl methacrylate (HEMA, 43.4 µL, 16 mol.%), LAP (6 mg), and 1474.4 µL deionized water. The enzymes GOx (14.05 mg/mL, lyophilized powder) and HRP (4.82 mg/mL, essentially salt-free, lyophilized powder) were dissolved in phosphate-buffered saline (PBS buffer, 100 mM, pH 7.4). Prior to the photopolymerization of the hydrogel, 50 µL of each of the enzyme solutions was mixed with 500 µL of the hydrogel precursor. The precursor was then exposed to a UV light intensity of 1 mW/cm² for 15 s in the microfluidic reactor chamber. After exposure, the microreactor was rinsed and filled with buffer solution and stored in the refrigerator at 8 °C.

**Figure 4:**
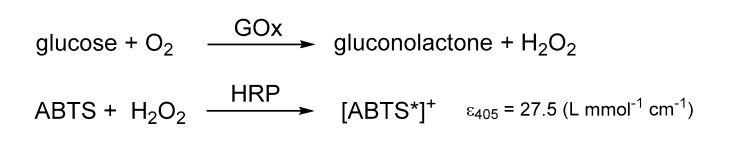
Bi-enzymatic cascade reaction with *Aspergillus niger* (GOx) and horseradish peroxidase (HRP).

To analyze the enzymatic activity of the hydrogel-immobilized enzymes in the microfluidic device, an assay reaction was performed, which could be quantified by UV–visible light (UV–Vis) spectroscopy. For this purpose, a substrate solution containing ABTS (5 mmol/L) and glucose (5 mmol/L) in PBS buffer (100 mM, pH 7.4) was pumped with a syringe pump (LA 300, Landgraf Laborsysteme, Langenhagen, Germany) through the microfluidic devices at a flow rate of 10 µL/min. The GOx and HRP catalyzed cascade reaction forms [ABTS*]^+^ which were detected and quantified by UV–Vis spectroscopy (Figure 4). The measurement was performed with a SPECORD^®^ 210 PLUS (Analytic Jena AG, Jena, Germany) spectrometer. More information about the hydrogels, the enzymatic cascade reaction, and the detection method was previously published [13,39].

## 3. Results

### 3.1. Photopolymerization of Hydrogel Patterns and Characterization

We focus on the production of reliable and functional hydrogel structures using the described lithography set-up. In the context of microfluidics, the main advantages of the free-radical UV polymerization from a monomer solution in a photolithography process are the easy handling, fast transfer of structures, and the compatibility of the technology within a microfluidic system. However, this also results in difficulties for micro structuring due to scattering in the solution and increased light refraction at the phase boundary from monomer solution to solid, oxygen content, and free diffusion of radicals during the exposure [30].

**Figure 5:**
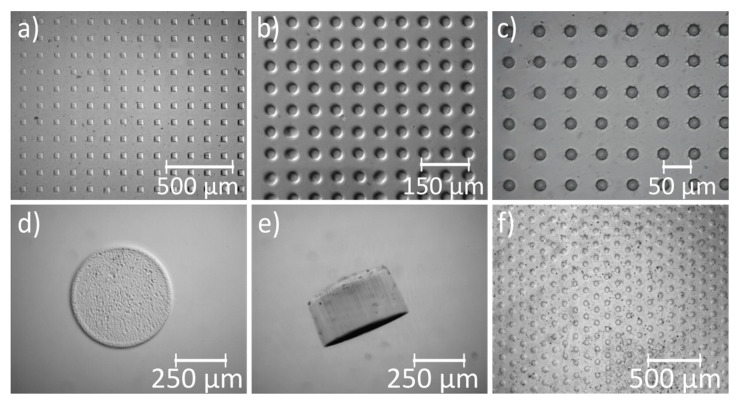
Hydrogels of different sizes polymerized by UV photolithography from a monomer solution: (**a**) swollen poly(*N*-isopropylacrylamide) (PNIPAAm) hydrogel cubes (50 µm) in array formation, as well as (**b**) 50 µm cylinders and (**c**) cylinders with diameter of 20 µm. (**d,e**) Top and side view of PNIPAAm gel cylinder (500 µm diameter, 250 µm height). (**f**) Arrangement of poly((ethylene glycol) diacrylate) (PEGDA) gel cylinders (50 µm diameter).

With the presented methods, flip-chip and in situ polymerization, angular and round structures of 50 µm footprint size (Figure 5a,b) were fabricated. For PNIPAAm, the smallest feature size shown here is 20 µm (Figure 5c). The smallest feature size of the of the mask resolution was limited to 10 µm. While smaller sizes than 20 µm could be polymerized, the quality of the structure resolution in this range decreased significantly and, thus, remained open for further optimization. Round hydrogel geometries were more advantageous to miniaturize. The formation of clear edges and vertical sides was tested on bigger geometries (Figure 5d,e), which also served to verify the parallel coupling of UV rays into the photoactive solution by the lithographic system. The smallest feature size of cylindric PEGDA gels presented here is 50 µm (Figure 5f).

**Figure 6:**
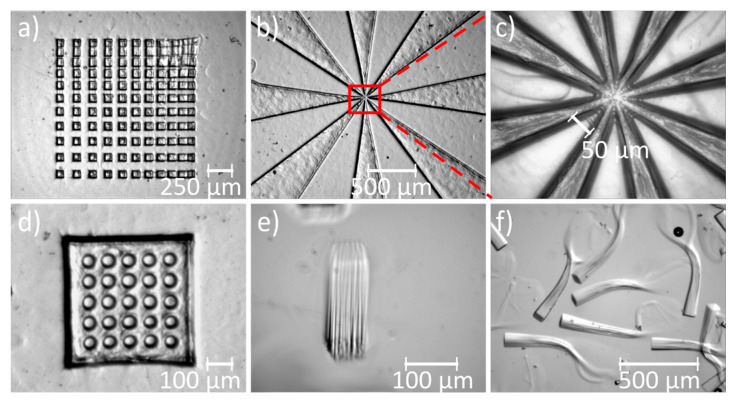
Hydrogel test structures for investigation of the patterning accuracy and transfer quality: (**a**) Integration-density test array with varying gaps (cubes of PNIPAAm dried, 50 µm side length). Siemens star test-structure (PNIPAAm dried) with (**b**) 16× and (**c**) 80× magnification. (**d**) Dried PNIPAAm layer with holes of 50 µm diameter. (**e**) PNIPAAm hydrogel post with 50 µm side length of square footprint and an aspect ratio of 1:5 (cavity height 500 µm, picture was taken at a tilting angle of around 45°). (**f**) PNIPAAm hydrogel post with 50 µm square footprint to achieve an aspect ratio of 1:10 in a cavity height of 1 mm, which was not successful.

Structuring tests regarding the integration density up to the performance limit of qualitatively acceptable hydrogel structures were detected for a structure gap of 50 µm. In Figure 6a, the distance between the hydrogel structures was iteratively reduced; beginning at a space of 100 µm, polymeric interconnections began to form between the hydrogel structures at a gap size of 50 µm. These interconnections could also be determined during the photopolymerization of a so-called Siemens star at 50 µm pitch and below (Figure 6b,c). As soon as interconnections between the hydrogels exist, they can no longer be seen as independently of each other and lose their designated function. However, this negative effect did not occur when we polymerized a hydrogel layer with holes of 50 µm diameter (Figure 6d). The reason for this was the reduced probability of free diffusing radicals due to the higher volume of the gel and the resulting low availability of diffusing radicals. To limit the effect of free diffusing radicals and the consequently formed interconnections, the amount of initiator should be reduced when exposing patterns in high densities [30]. We polymerized hydrogel posts of 20 µm diameter at a gap of 50 µm, which corresponds to 2 × 10^4^ hydrogels per cm². The hydrogels patterned here can be activated independently by heat induction (e.g., transferred by opto-electrothermic controllers [15]) or can be used as storage elements for chemical information [11] (see Section 3.3). Thus, the degree of integration for the hydrogel patterns produced here was classified as suitable for VLSI, based on the technical term for the degree of integration of more than 10^4^ active components per chip in analogy to microelectronics.

The aspect ratio is another important benchmark for quantifying the manufacturing method of integrated structures. It could be shown that, with nano-imprinting, aspect ratios of hydrogel geometries of 1:12 and a very high density of more than 4.4 × 10^7^ pillars per cm² were achieved reproducibly [23]. However, this requires a micro-structured stamp and a time-consuming process. With our technological approach, which is qualified to be fast, versatile, flexible, and cost-effective to polymerize hydrogels from a solution, we obtained hydrogel posts with an aspect ratio of at least 1:5 (Figure 6e). As the aspect ratio increased, the diameters of the structures decreased due to adsorption of the UV light in the hydrogel bulk and at phase boundaries. For the same reason, hydrogels with high aspect ratios become slightly trapezoidal. During an attempt to achieve an aspect ratio of 1:10, it was observed that the hydrogel posts split into several polymer strands resembling tentacles (Figure 6f). This was most likely due to the dispersion and scattering of UV-light through the already polymerized hydrogel, which led to an inhomogeneous growth of the gel. It was observed that hydrogel posts fell aside for aspect ratios greater than 1:5, especially when shear forces occurred. This could be avoided by covalently binding the pillars to the substrate surface or by the use of supporting structures.

### 3.2. Comparison of the Methods for Hydrogel High-Density Integration

A major challenge of the flip-chip process is to ensure sufficient cleanliness for the bonding process without destroying the hydrogels by the plasma treatment or evaporation. The oxygen plasma bonding can be critical for hydrogels with biological components, such as immobilized enzymes, due to the vacuum and plasma treatment, which can lead to drying out or bursting of the hydrogel structures. Moreover, with increasing integration level and miniaturization, the alignment of hydrogel patterns to microfluidic structures becomes more and more difficult [29,37]. The shrinkage of PDMS during baking and the resulting discrepancy will lead to misalignment. For this reason, either the position of the hydrogels for the mask design should be placed in a relatively narrow space to ensure sufficient tolerance later on or steps for the reduction of the PDMS shrinkage have to be performed by reduced curing temperatures, using predictions in design [36], or by an additional rigid layer sandwiching the PDMS [40]. During the flip-chip process, there is also a higher risk that hydrogels will detach from the substrate material during disassembly of the polymerization chamber after exposure or while rinsing. Further miniaturization or higher hydrogel aspect ratios increase the risk of losing hydrogels during transfer. The covalent attachment of hydrogels to the substrate surface by a pre-treatment is an approach to overcome this problem [41]. Nevertheless, the flip-chip method enables fast and easy reproduction of hydrogels with high integration densities. Furthermore, the lithographic process allows simultaneous production and integration of different hydrogel-based components [14]. Moreover, it is possible to produce hydrogels with different heights by selecting the appropriate spacer height of the cavity during the polymerization process. The flip-chip process is the preferred method for stacking different multifunctional layers for a 3D microfluidic chip architecture, as each layer can be functionalized independently and is then aligned and joined with others (layer-by-layer technology). Additionally, the separate fabrication of hydrogel structures outside the channels and cavities allows a simple definition of the ratio of the (dry) volume of hydrogel structures and the volume of a cavity, e.g., a valve chamber, as is required for valve design with well-defined opening or closing behavior. A good alignment system is, therefore, a basic requirement to be able to use the flip-chip process to its full extent for the production of highly integrated hydrogel systems.

In contrast, one advantage of the in situ polymerization over the flip-chip procedure is that there are fewer critical alignment steps involved. When it is needed for a specific application, the in situ polymerization ensures that the hydrogel geometry exactly fills the microfluidic channels in the vertical dimension. In addition, there are fewer influences of radical scavengers such as oxygen and contamination since the polymerization occurs in a closed system. Moreover, there is no harm by vacuum or plasma treatment on the reactive monomer solution during and after polymerization, since the polymerization is the last step of the process. One challenge with this procedure is to bring the mask very close to the microfluidic channels to reduce scattering and shadowing. The in situ polymerization of hydrogels inside a 3D chip architecture of different layers is not recommended due to scattering and adsorption of the UV light. Furthermore, the occurrence of bubbles during filling of the monomer solution in the system will greatly affect the success of the process. Therefore, increased effort during microfluidic chip design is needed to reduce the enclosure of gas bubbles and improve flow and filling distribution [42]. Utilizing rounded corners, conical-shaped inflows and uniform resistances of parallel flow paths by symmetric geometries during the chip design will improve homogeneous filling later. By cooling the backside of the chip, for example, by placing it in a cold water bath during exposure, the thermal overload in the polymerization chamber by the UV source and reaction energy is prevented. Thermal overload during in situ polymerization is more critical than in the flip-chip method since heat cannot be dissipated well in enclosed polymer structures. The selection of appropriate materials dominates the in situ process. The materials must be suitable for the fabrication of microfluidic structures and for chip bonding. In addition, they must be available as thin and stable layers with high transparency and UV transmission. Therefore, the selection of available materials is reduced to a few elastomers (e.g., PDMS and polyurethane) and thermoplastics (e.g., poly(methyl methacrylate), cyclic olefin polymers, and cyclic olefin copolymers), as well as glass. In summary, the flip-chip method offers a high degree of freedom in design and materials paired with the simultaneous integration of different hydrogel-based components on a microfluidic chip in one step. On the other hand, the in situ polymerization allows higher precision and reproducible formation of gels into a microfluidic chip. Additionally, it leads to less contamination and waste of the monomer solution during chip preparation. Nevertheless, both technologies are combinable with established processes for microsystem manufacturing with commonly used materials and offer a high degree for miniaturization and mass production of hydrogel-based microsystems in the future [38,43].

### 3.3. Hydrogel-Based Microreactors: Increasing Yield by Miniaturization

In the field of (micro-)electronics, the miniaturization of integrated circuits and its components, which ensures faster data processing and lower resource consumption, was long a driver for research and innovation (Moore’s law). The miniaturization of microfluidic systems comparably leads to significant advantages in terms of reaction rate, throughput, and reduced resource consumption by parallelizing tasks and increasing the integration density of microfluidic structures. One challenge is that detection methods and the analysis of reagents in volumes from micro- to nanoliters must be provided, e.g., by higher output rates in a scaled-down LoC system [44].

In previously published research studies, PDMS-on-glass microfluidic devices with hydrogel structures were developed, and enzymes were physically entrapped in hydrogels [13,14,45,46]. Miniaturization of those devices would have been the logical next step, e.g., by increasing the surface area to volume (SA:V) ratio in the reaction chamber.

Based on the improved parameters for the photopolymerization technique of hydrogels with the developed UV irradiation set-up, it was aimed to apply the technology for the miniaturization of enzyme-containing hydrogels (hydrogel-enzyme-pillars) from a diameter of 350 µm down to 50 µm in microfluidic devices.

#### 3.3.1. Design of the Microfluidic Device

Here, we want to utilize the developed photolithography set-up for the precise structuring of hydrogels with the in situ process to benefit from the advantages of this method (see Section 3.2). In contrast to our previous study, the photostructured hydrogels obtained with the in situ process possessed the same height as the microfluidic chamber and were, thus, called pillars instead of dots. Moreover, the method enabled the patterning and investigation of thinner hydrogel–enzyme pillars that are not accessible with the flip-chip process due to fast drying of the gels. Consequently, the in situ process allows performing further investigations on the enzymatic microreactors such as the dependence of the enzymatic conversion on the SA:V ratio of the hydrogel pillars within the device. As it was shown before [14], the immobilization of the enzymes in the hydrogel matrix slows down the conversion of the substrates as their diffusion toward the enzyme is decelerated. It is, therefore, assumed that an increase in SA:V ratio of the hydrogel pillars promotes the catalytic conversion of the substrates.

To investigate the conversion in microfluidic devices and the relation between the SA:V ratios of hydrogel matrices, microfluidic devices containing PEGDA enzyme pillars with different diameters (350 µm (R1), 150 µm (R2), 50 µm (R3)) were produced in cavities of 150 µm. In this way, the total volume of the hydrogel in the microreactors was kept constant, *i.e.*, with decreasing diameter of the pillars, their number was increased. Consequently, both the amounts of immobilized enzymes and the residence time of the fluid in the device were the same for each microreactor type. At the same time, the SA:V ratio increased with decreasing pillar size. For example, in R3, the hydrogel pillars had a sevenfold greater SA:V ratio compared to R1 (Table 1). In all cases, the hydrogel pillars were arranged hexagonally, as previous investigations revealed the increased contact between hydrogels and fluid, which promotes catalytic conversion of the enzyme substrates dissolved in the fluid [13].

The enzymes GOx and HRP were immobilized in the microfluidic devices R1–R3, as their catalytic activity under a continuous flow of the substrates ABTS and glucose could be directly measured by UV–Vis spectroscopy and the functionality of the design could, therefore, be easily visualized. In the two-step enzyme-catalyzed conversion, glucose is firstly oxidized to gluconolactone by GOx, whereby H_2_O_2_ is formed. Subsequently, HRP catalyzes the conversion of ABTS to [ABTS*]^+^ upon consuming H_2_O_2_ (see Figure 4). Consequently, the concentration of the formed [ABTS*]^+^ and, therefore, the absorption measured by UV–Vis spectroscopy depends on the catalytic activity in the device, which is determined by the amount of immobilized enzymes, the efficiency of the immobilization, and the accessibility of the enzymes. Apart from that, the conversion clearly depends on the flow rate of the substrate solution, as the residence time of the substrates at the hydrogel–enzyme pillars linearly decreases with increasing flow rate. When the parameters for the photopolymerization of the hydrogel–enzyme pillars are appropriately set, reliable enzyme immobilization can be obtained, which results in stable long-term conversion of the substrates pumped through the microfluidic devices.

**Figure 7:**
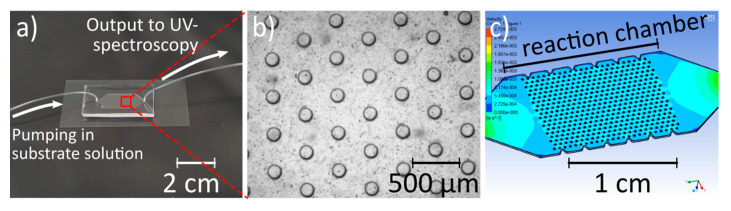
(**a**) Photograph of a polydimethylsiloxane (PDMS)-on-glass microfluidic device with hydrogel–enzyme pillars. The inlet and outlet are connected to tubes through which the substrate solution (2,2′-azino-bis(3-ethylbenzothiazoline-6-sulfonic acid) diammonium salt (ABTS) + glucose) is pumped into the device and transferred to the flow cuvette for UV–visible light (UV–Vis) spectroscopy. (**b**) Hydrogel–enzyme pillars with 150 µm diameter and 150 µm height (exposure time 15 s). (**c**) Exemplary simulation of fluid velocity and flow distribution for the microreactor with the respective hydrogel–enzyme pillars. The dimensions of the reaction chamber are 13 mm in length and 8 mm in width.

An exemplary photograph of a microfluidic device and the schematic measurement set-up is shown in Figure 7a,b. A flow distribution simulation for each microreactor type was performed (Figure 7c). In this way, it was shown that the fluidic resistance for the different reactors and, thus, the pressure did not significantly change, as the cross-section of the reaction chamber and the total hydrogel volume remained the same. Regardless of the pillar size, the flow was uniformly distributed at the applied flow rate (10 µL/min), and spatial contact with the hydrogel pillars was achieved. Consequently, the results of the absorbance measurement obtained with the different types of microreactors were comparable with one another with regard to the flow characteristics. Variations of the absorbance can, thus, be attributed to the varying SA:V ratio. However, it has to be noted that the leakage of the enzymes from the hydrogel–enzyme pillars was probably size-dependent. In the case of smaller pillars, a higher proportion of the enzymes was located on the outer part of the pillars, which might lead to increased leakage and, thus, to decreased catalytic activity within the microreactor.

#### 3.3.2. Enzymatic Conversion in Dependence of SA:V Ratio: Optimization and Results

**Figure 8:**
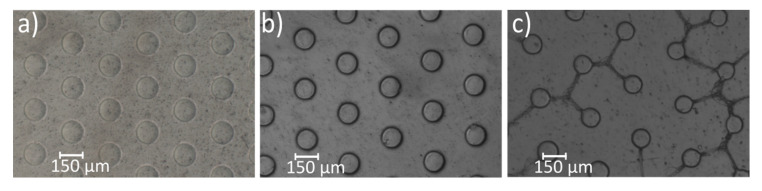
Microscope images of hydrogel–enzyme pillars polymerized with a mask diameter of 150 µm and cavity height of 150 µm. The exposure times were (**a**) 10 s, (**b**) 20 s, and (**c**) 30 s.

In initial experiments, hydrogel–enzyme pillars were photopolymerized with an exposure time of 10 s and applied for the flow-through measurements of the catalytic activity. However, in contrast to our previous investigations with GOx- and HRP-containing hydrogel pillars, no constant substrate conversion was achieved, and the conversion was generally very low [13,14]. Apart from that, a high absorbance was detected in the beginning of each measurement. From these results, it was concluded that no reliable enzyme immobilization was achieved, and that the enzymes were washed out from the insufficiently cross-linked hydrogel matrix. Therefore, a high substrate conversion was initially promoted by the no longer immobilized enzymes, which were subsequently washed away from the device. Because of these considerations, the irradiation time was increased to 20 s in a next step. This led to a higher contrast and a lower diameter of the hydrogel pillars after 10 s of exposure time (Figure 8a,b). Both aspects visualized the increased degree of cross-linking, which likely prevented the leakage of the enzymes from the hydrogel–enzyme pillars. Further increase of the irradiation time to 30 s was not useful, as clearly separated hydrogel pillars were no longer obtained (Figure 8c). Instead, the pillars were interconnected with one another, caused by overexposure of the precursor solution (see Section 3.1), which hindered the fluid flow in the microfluidic device and resulted in dead zones.

**Figure 9:**
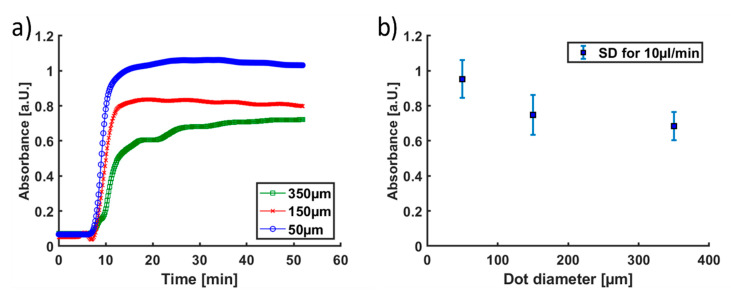
(**a**) Exemplary flow-through measurement of three microfluidic chips containing hydrogel-enzyme pillars with GOx and HRP. Substrate concentration (ABTS and glucose): 5 mmol/L; flow rate: 10 µL/min; residence time of the substrate in the microfluidic device: 3.9 min. The offset (10 min) of the time-dependent absorption results from the offline measurement and time required for filling the microfluidic tubes and the flow cuvette. (**b**) Absorbance values measured at the absorbance plateaus (30–60 min measurement time) in dependence of the diameter of the hydrogel–enzyme pillars.

As the first experiments indicated, the measurement curves of the time-dependent absorption of longer exposed structures showed a significantly higher enzyme immobilization compared to the results with the shorter exposed hydrogel pillars (Appendix C). However, with extended exposure times, the risk of interconnections (Figure 8c) increased, whereby the reaction chamber was no longer uniformly flooded, and the reproducibility of the output was reduced. Consequently, the conversion in microfluidic devices with hydrogel–enzyme pillars polymerized with an exposure time of 15 s was chosen as standard parameter, as this was the best trade-off between sufficient polymerization times for the different pillar diameters without inhomogeneities. Constant, *i.e.*, time-independent, absorbance values were obtained for all types of microfluidic devices after equilibration of the system (Figure 9).

Thus, the conversion within the microfluidic chip R1 is in accordance with the previously obtained results with the same hydrogel pillar size [14]. Consequently, these results allow deriving the relationship between the SA:V ratio of the hydrogel pillars and the substrate conversion. As Figure 9a shows, the highest conversion rate was obtained with the smallest diameter of the hydrogel pillars (50 µm). However, within the standard deviation, the conversion with 150 µm and 350 µm did not change much (Figure 9b). Thus, the increase in the SA:V ratio of the hydrogel pillars within the investigated range resulted in a lower increase of the conversion than theoretically predicted (Table 1). Everything indicates that this observation can be attributed to the above-mentioned increased leakage of the enzymes from the smaller hydrogel–enzyme pillars which partly counterbalanced the benefit of the increased SA:V ratio. Nevertheless, it was shown that, with the herein applied methods, the enzymatic conversion within microfluidic devices could be enhanced by decreasing the diameter of the hydrogel–enzyme pillars from the previously used 350 µm to 50 µm, thereby increasing the SA:V ratio.

## 4. Conclusions

In this paper, a technology suitable for the production of VLSI-ready hydrogel structures by photolithographic process in microfluidic systems, ranging to the lower micrometer scale, was presented. Photopolymerizing hydrogels in an aqueous solution has its challenges, e.g., scattering effects and control of the free diffusion of radicals. At the same time, the presented methods harmonize strongly with microfluidic concepts, as the monomer solution can be injected in chips, and fluidic channels are used for system depended adjustment. Due to low processing times and harmless production conditions, biocompatible systems can be produced on different substrates at high throughput by photolithography. The success largely depends on the chemical compounds and the mask quality, as well as the intensity, exposure time, and homogeneity of a parallelized UV light source. In this paper, we presented a self-designed, low-cost photolithographic set-up that combines a UV LED light source and a system of various lenses for beam parallelization and homogenization. A flattop profile with a homogeneity of 98.98% was achieved. The opportunity to tune the LED to the desired performance characteristics and the free adjustment of the optical components based on a calculated optimized beam path gave the opportunity to adapt the photolithographic system to various applications for microfluidics and the polymerization of various hydrogels (PNIPAAm and PEGDA). We used mask-based photolithography for two different methods (flip-chip and in situ polymerization) to structure hydrogels and discuss their impact on development of highly integrated microfluidic systems. Both methods led to reproducible structures down to 20-µm resolution at a 50-µm pitch (20,000 per cm², suitable for VLSI) and an aspect ratio up to 1:5.

Overall, the method of the in situ polymerization of hydrogels was successfully applied for the production of microfluidic devices with hydrogel–enzyme pillars. The exposure time was tuned to 15 s to achieve sufficient cross-linking of the hydrogels and, thus, reliable enzyme immobilization while preventing interconnections between the hydrogel pillars due to overexposure and blocking of the fluid flow. Based on the obtained results, a gain of the conversion by increasing the SA:V ratio was derived (at least 40% for scaling 350 µm pillar diameter to 50 µm). Consequently, the polymerization of hydrogel pillars with the in situ method is a valuable addition to the previously used flip-chip method to achieve further miniaturization in microfluidic devices and to intensify catalytic processes. However, it has to be noted that the benefit of an increased SA:V ratio might be stronger for other applications. For example, in the case of covalent enzyme immobilization on the outside of the hydrogel pillars, an increased SA:V ratio will result in a higher amount of accessible catalysts, thus promoting the substrate conversion.

The application of enzyme-containing hydrogel pillars presented here was used to demonstrate the advantages of miniaturization and resulting potential problems (e.g., increased leaching effects by a higher SA:V ratio). With the presented photolithography set-up and the different polymerization methods, it will be possible to establish various hydrogel systems with similar integration densities to those shown here. In principle, any application of hydrogels in array formation, from haptics to different LoC technologies for protein or DNA immobilization, can be miniaturized and, thus, be made more effective.

## Figures and Tables

**Table 1:** Geometries of the microreactors R1, R2, and R3 with different diameters of the hydrogel–enzyme pillars and consequently different surface area to volume (SA:V) ratios. The total surface area (SA) results from the number of pillars multiplied by the lateral surface of the hydrogel pillar.

Reactor	Pillar Diameter (µm)	Reactor Height (µm)	Number of Pillars	Total Hydrogel Volume (µL)	Total SA of Hydrogel (mm²)	SA:V Increase Relative to R1
R1	350	150	104	1,51	17.3	1.0
R2	150	150	570	1,51	40.3	2,3
R3	50	150	5132	1,51	120.9	7.0

**Figure A1:**
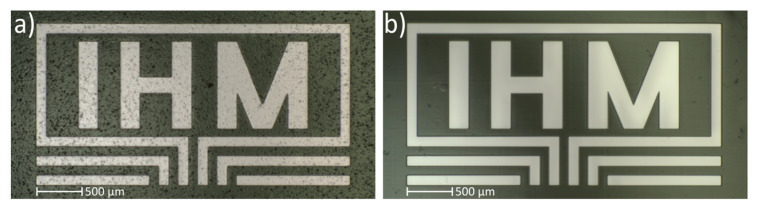
Comparison of the quality of a polymer mask (**a**) and that of a glass mask (**b**). The polymer film has an inhomogeneous surface and enclosures, which lead to more shadow and scattering effects compared to a glass mask.

**Figure A2:**
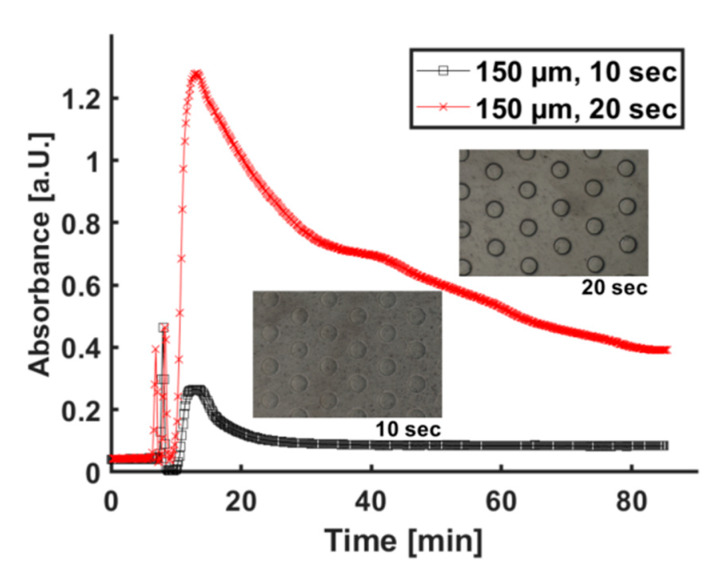
Measurement of the 150 µm type (R2) to investigate the leaching effect of enzymes from PEGDA gel dots with different exposure times (10 and 20 s). Here, the amounts of enzymes (U) in the solution had to be reduced to one-third (50 U/mL) of the standard recipe to not exceed the measurement scale of the UV–Vis spectroscopy.

## Appendix A

### Fine-Tuning the UV Exposure System

The implementation of a high-quality UV lithography system for the photopolymerization of gels is beneficial because it is needed for other microstructure processing techniques as well (e.g., master production for the soft lithography with PDMS). To ensure defined structured hydrogels with good reproducibility and, at the same time, to miniaturize them in order to increase reaction speed and actuator density, the demands of such a photolithography system are high. We decided to develop our own photolithography for structuring hydrogels instead of buying an expensive set-up. The exposure system includes a light source with controllable intensity, high parallelism, and homogeneity for an exposed area of four inches (maximum wafer size of the standardized microfluidic systems used). Furthermore, the process handling, the environmental conditions, and parameters during the exposure should be precisely tunable to the sensitivity of biomaterials and chemical components. Therefore, we use UV LED illumination as a cost-efficient, monochromatic light source. In practice, UV LEDs offer most of all high reliability, simple control, relative insensitivity to optical feedback due to thermal backside cooling, instant use at full power potential, and increased power efficiency in contrast to other sources like halogen mercury vapor lamps. The use of a LED array instead of a single high-power LED favors a homogeneous illumination of a specific area. LED-based systems are easily scalable and interchangeable if another wavelength range is needed [47,48].

Increased effort is required for cooling the LED array, firstly, to increase lifetime of the semiconductors and, secondly, to prevent heat-induced wavelength shift (a temperature rise at the pn-layer of about 10 K corresponds to a shift by 1 nm light wavelength and a slight decrease in intensity with increasing temperature over time [48]). For the design of the lithography system, different optical and mechanical components were selected. In order to gain first practical insights, it was validated how the light of the UV source can be directed with the different lens types with regard to homogenization and parallelization, and which power loss through the lens system has to be calculated. Later, specific optical components were chosen, and a first prototype was modeled in the simulation program (Winlens 3D, Qioptiq Photonics GmbH & Co. Kg, Göttingen, Germany) for parameter optimization. For the collimator, we use an aspheric condenser lens with diffuser surface, which has a positive effect on the uniformity, but also reduces the light intensity. Since we decided to use a high-power LED, there are no restrictions with regard to insufficient luminous efficacy. All used lenses are coated with UV antireflective.

## Appendix B

### Optimizations for the Polymerization Process

High-quality photolithography is characterized by a sufficient parallelization of the UV exposure beam, an optimal mask transmission, and a homogeneous light field for a reproducible control of the light intensity on the imaging plane. Good masks and the control over process parameters reduce unwanted scattering, shadow effects, and slanted profiles. Contact lithography is recommended for the exposure of all used photosensitive structures with the presented lithography set-up. Masks can be fixed generally very well on the sticky surface of PDMS without any further aids. For other substrate materials, it is recommended to put a drop of water between mask and chip to achieve a fixation by applying even planar contact pressure without having an air gap. If it is compatible with the materials, immersion oil can also be used for improved transmission with reduced refraction angle. The photolithographic system should be tuned to the desired wavelength of the initiator to make the formation of radicals effective. The exposure time and light intensity can be found in product data sheets of the initiator and in the literature, but they usually require fine-tuning depending on the equipment, microsystem material, and conditions of use. For UV photopolymerization in aqueous solutions, there is also the problem that radicals with a certain path length diffuse freely and initiate polymer chains in unexposed areas. This problem can be countered by reducing scattering effects, reflection, exposure time, and intensity [30].

Scattering effects are reduced by the smallest possible distance between mask and substrate, as well as by materials with high UV transmission. Substrates placed between the mask and the polymerization chamber prevent the adhesion of hydrogels to the mask and their contamination, but also influence the structure transfer depending on layer thickness, scattering, reflection, and the material-specific absorbance of UV light. The use of a thin glass slide is superior to plastic substrates due to its better UV light transmission. It is also common to use PDMS as a monolithic chip substrate for prototyping because of the easy and fast handling of the process and its relative high UV light transmission (see in situ polymerization in Section 2.4.2). However, there are other polymers, especially thermoplastics, that are also suitable for the hydrogel integration methods presented here, which should be considered when dealing with other aspects than prototyping, since PDMS is not well suited for final microfluidic chip production [49,50]. Low reflective substrates for the polymerization chamber and a black background surface will significantly improve the results.

For a reliable structure transfer at relatively low cost, we prefer glass masks to plastic masks due higher quality, and we favor them over the much more expensive chrome masks, which only become necessary in the sub-micrometer range (Figure A1). For polymerization below the lower critical solution temperature of PNIPAAm (32 °C), as well as keeping controlled conditions of reaction heat, the backside of the polymerization chamber should be cooled in a water bath before and during the exposure. Hydrogels that were polymerized on a glass substrate usually tend to adhere on the surface. In addition, if hydrogels adhere to a surface, the drying causes cracks in the macroscopic structure, due to unconformable shrinking. Dried hydrogels have a higher deviation in their stability compared to hydrogels that are kept humid all the time. Furthermore, due to an enlarged surface, some cracked hydrogels show an increased degree of swelling. For reliability purposes, the rapid drying of hydrogels is avoided to prevent cracks. The storage of prepared hydrogels in chilled water in the refrigerator (8 °C) does not affect their durability.

It should also be mentioned that hydrogels swell from the defined size in the production state to a larger form afterward (swollen state). For the PNIPAAm recipe presented here, we determined a post-swelling of 10 %. Depending on the degree of cross-linking and additives, this can be respectively smaller or larger. The physical properties of the hydrogel (tensile strength, Young’s modulus) are defined by the used monomer (composition, concentration), the cross-linking system (mesh size, physical and chemical connections), and the chain length. A cross-linker system other than the commonly used BIS are nanocomposite clays such as Laponite XLS (BYK-Chemie GmbH, Wesel, Germany), which improve the mechanical properties in terms of tensile strength and elongation at break, but also increase the scattering of UV light during polymerization and reduce the swelling behavior, depending on the content of added nanoparticles [51]. The gel cylinder in Figure 5d and e was prepared with PNIPAAm and clay. In addition to the cross-linker and additives, the physical stability of gels is supported by the degree of polymerization. The chain length can be controlled by increasing the statistic initiation of radical polymerization reactions, availability of monomers, and the probability of chain termination reactions. The choice of the UV initiator, its reactivity and solubility, and the intensity, duration, and wavelength of the emitted light spectrum will also influence the structure and material properties of the hydrogel [52]. To achieve a higher degree of swelling of PNIPAAm, small doses of sodium acrylate (1–5 mol.%, Sigma-Aldrich, St. Louis, MO, USA) may be added [19]. However, there are far more compositions and additives that support the future prospects of hydrogels by giving them multi-functionality.

## Appendix C

### Challenges Due to Miniaturization: The Leaching Effect

The leaching of enzymes led to unexpected results, especially with very small structures with increased SA:V ratios. For this reason, the effect of leaching was investigated more closely. The hydrogel–enzyme pillars were polymerized with a UV-intensity of 1 mW/cm² for 10, 20, and 30 s. The highest exposure time led to channel blockage and was, therefore, not suitable. The comparison of shorter and longer exposure times (10 and 20 s) showed that the absolute absorption of the [ABTS*]^+^ produced was significantly higher and the washout of enzymes was slower with longer exposure times over time and vice versa for shorter exposure times (Figure A2). Longer exposure times led to a more strongly cross-linked gel-structure and, thus, to a better immobilization of the enzymes. This assumption is supported by the corresponding images of the differently exposed hydrogels (from Figure 8a,b) next to the respective curve. The hydrogels exposed for 20 s showed pillars with significantly higher contrast. In contrast, pillars with 10 s of exposure time were more transparent, had rounded edges, and were slightly larger in diameter. The difference in size can be explained by a less strong polymer network, which swelled more after production in solution and should, therefore, have a higher diffusion coefficient.
